# Facts Confirmatory of the Value of Vaccination

**Published:** 1840-07

**Authors:** 


The following interesting facts were communicated to the editor of the Boston Medical and Surgical Journal, by Dr. Silas Brown, of Wilmington.

### FACTS IN RELATION TO VACCINATION.

I submit the following statement of such facts as have recently come under my observation, hoping that they may prove useful in convincing the skeptical of the efficacy of kinepox as a substitute for small pox. Many reports have been in circulation among the people, in this vicinity, detrimental to the expansion of cowpox, which have caused a delay of vaccination till the smallpox has broken out in some of their families. One is, that it will serve only a temporary purpose; and unless re-vaccinated, a person is as liable to have the smallpox after vaccination as before. Another is, that it only answers a partial purpose, and will not wholly insure a person against variola or varioloid, and therefore he may through life live under continued apprehensions and fears of the contagion of those varieties of the same disease.

It is not my object to give a detailed account of the treatment of my patients, but merely to show that the cowpox has been an effectual antidote to smallpox in the several instances to be described.

About the first of February last, the smallpox made its appearance in four different families, in one of our school districts; the subjects were four scholars; the first a lad about 18 years old, the second a girl about 12, the third a girl of 9, the fourth a boy about 8. Neither of them had ever been vaccinated. The family to which the first belonged consisted of four members, all adults, besides the patient, two of whom had been vaccinated and two had not. They were all vaccinated as soon as the eruption gave satisfactory evidence that the patient had the smallpox. It proved a severe case; the face was so swollen that the patient's eyes were closed for several days. The eruption appeared on the 4th day of February and ran through its course without a secondary fever, and he speedily recovered his health. Now it is the 4th day of May, and neither of the vaccinated persons has suffered any sickness, except what follows vaccination.

The second broke out on the 5th day of February, and a similar case to the first, till the 13th, when the secondary fever took place, accompanied with cough and inflammation in the lungs, which continued till the last of the month before her fever manifested any abatement, and her friends despaired of her recovery. She is now restored to health, May 4th.—The girl lived in the house with her grandmother, uncle, aunt, and a large family of cousins. The uncle, aunt, and several of the cousins had never been vaccinated till two days after the eruption appeared upon the patient, when the whole family were vaccinated, and have suffered no inconvenience, except from vaccination, to the present time, May 25th.

The eruption upon the third patient appeared on the 5th of February, was of the distinct kind, and ran a regular course. The secondary fever was slight, and the patient recovered without any distressing symptoms. The parents of this child had been vaccinated nearly 30 years, and part of the children about three years. The remainder I vaccinated two days after the eruption appeared upon the patient. The youngest, a child about two years old, kept with its mother, who had the care of the patient, night and day. About the 11th day after vaccination, the child had a sore arm and other symptoms accompanying cowpox, and appeared more feverish than the other children vaccinated at the same time. On the 14th day from the eruption of smallpox on the other patient, the infant broke out with varioloid, which passed off in a very mild manner, and was the only case of varioloid which occurred among the numerous members of the above-stated families. Is it not probable this child had received the contagion previous to vaccination?

The fourth patient broke out on the 7th of February. His disorder ran a regular course, and terminated favorably. The family consisted of four adult persons and one infant. The mother of the patient and the infant had not been vaccinated previously to the sickness of the patient, but they were soon afterwards, and the other members were re-vaccinated at the same time. Now it is the 25th of May, and none of them have suffered any sickness, except what is consequent to kinepox.

I was called to a child the 8th of April, in a neighboring town, about six months old, with an eruption which proved to be smallpox of the distinct kind, and which ran a favorable course. The parents of the child and other children of the family had been vaccinated. The mother nursed the child to the close of the disorder, and none of them suffered any inconvenience from contagion.

I think, in the above stated cases, the evidence adduced must convince the doubting part of the community, and amply testify to the efficacy of cowpox as a substitute for smallpox. The people in this vicinity have dismissed their doubts which formerly existed, and have full confidence in the virtues of vaccination.

